# Supplementary material for: Epidemiological Profile and Risk Factors for Malaria in Rural Communities Before the Operationalization of the Singrobo–Ahouaty Dam, Southern Côte d’Ivoire
Source: Trop Med Infect Dis. 2025 Jul 15;10(7):197. doi: 10.3390/tropicalmed10070197 (PMC12300484; doi:10.3390/tropicalmed10070197)
Supplement: Supplementary file 1 [file tropicalmed-10-00197-s001.zip › Table_Covariate_for Risk factors.pdf]

Table S1: List of 40 questions administered to household heads in the villages in 2021.

Covariates	Modalities	N	n (%)	OR (CI 95%)	p
Religion	#No belief	9	3 (33.3)		0.809
	Animist	3	1 (33.3)	0.6 (0.1 – 7.9)	0.701
	Christianity	167	41 (24.6)	0.2 (0 -10.4)	0.446
	Islam	65	24 (36.9)	0.5 (0.1 – 2.5)	0.398
Educational statute	#Higher education	8	1 (12.5)		0.017*
	Secondary	75	13 (37.1)	0.2 (0 – 4.9)	0.320
	Primary or Koranic school	35	14 (18.7)	1.9 (0.4 – 9.0)	0.889
	No schooling	126	41 (32.5)	0.2 (0.1 – 0.7)	0.156
Ethnic Group	#Indigenous	155	29 (18.7)		0.881
	Ivorian migrants	29	11 (37.9)	1.4 (0.3 – 7.8)	0.706
	Foreign migrants	60	20 (33.3)	1.5 (0.3 – 8.1)	0.645
Occupation	#No occupation	11	5 (45.5)		0.182
	Public sector employee	103	31 (30.1)	11.8 (0.6 – 237.1)	0.107
	Informal sector	70	20 (28.6)	19.4 (1.7 – 222.7)	0.017*
	Raw material sector	10	2 (20.0)	12.8 (1.0 – 159.0)	0.048*
	Market gardening	50	11 (22.0)	32.7 (1.1 – 962.6)	0.043*
Monthly income	#60 000 FCFA and more	51	9 (17.7)		0.074
	Less than 60.000 FCFA	134	46 (34.2)	0.1 (0 – 0.9)	0.037*
	No income	59	14 (23.7)	0.3 (0 – 2.2)	0.216
House ownership	#Tenant	227	65 (28.6)	1.7 (0.2 – 12.5)	0.618
	Owner	17	4 (23.5)		
Number of rooms in the house	#1–2 rooms	162	40 (24.7)	0.5 (0.2 – 1.4)	0.193
	> 2 rooms	82	29 (35.4)		
Number of people in the household	#One people	67	15 (22.4)		0.432
	2–5 people	74	17 (23.0)	0.9 (0 – 29.5)	0.949
	6–8 people	99	34 (34.3)	0.6 (0 – 20.0)	0.795
	> 8 people	4	3 (75.0)	1.6 (0.1 – 50.0)	0.801
Building material	# Hard	96	23 (24.0		0.890
	Semi-hard	68	18 (26.5)	0.8 (0.2 – 2.5)	0.644
	Clay/Banco/Wood	80	28 (35.0))	0.8 (0.2 – 2.6)	0.702
Knowledge of malaria	#No	241	68 (28.2)	0.6 (0 – 170.3)	0.843
	Yes	3	1 (33.3)		
Causes of malaria	#Mosquitoes	192	53 (27.6)		0.389
	Watercourses	6	2 (33.3)	1.6 (0.3 – 7.5)	0.565
	I don't know	46	14 (30.4	0.2 (0 – 5.4)	0.342
Sources of knowledge about malaria	#I don't know	188	51 (27.1)		0.880
	Word of mouth	34	10 (29.4)	0.3 (0 – 8.0)	0.483
	Television/Awareness	18	7 (38.9)	0.4 (0 – 15.6)	0.639
	Doctor/Nurse	4	1 (25.0)	0.3 (0 – 10.6)	0.471
Malaria symptoms	#Ignorant people	52	16 (30.8)	1.1 (0.3 – 3.3)	0.933
	People who know	192	53 (27.6)		
Are mosquitoes a nuisance?	#No	7	0		0.342
	Yes	4	66 (28.3)	0.1 (0 – 2.3)	0.143
	I don't know	233	3 (75.0)	0	0.999

Mosquito-borne-disease	#Malaria	32	12 (37.5)		0.754
	Other illnesses	3	1 (33.3)	0.9 (0.2 – 4.6)	0.858
	Malaria and other diseases	14	3 (21.4)	6.6 (0.2 – 210.4)	0.287
	I don't know	195	53 (27.2)	1.0 (0.1 – 8.5)	0.976
Easy access to the hospital	#No	234	65 (27.8)	1.1 (0.1 – 10.7)	0.950
	Yes	10	4 (40.0)		
Distance village-dam	#Less than 3 kilometers	98	30 (30.6		0.216
	3–5 kilometers	55	14 (25.4)	7.7 (0.3 – 207.7)	0.222
	6–10 kilometers	69	22 (31.9)	17.9 (0.8 – 399.2)	0.068
	More than 10 kilometers	22	3 (13.6)	9.1 (0.5 – 181.8)	0.148
Household illnesses	#Other	160	41 (25.6)		0.889
	Anemia	2	1 (50.0)	1.1 (0.2 – 7.8)	0.903
	Ulcers	5	1 (20.0)	1.7 (0 – 120.7)	0.798
	Malaria and other diseases	64	19 (29.7)	7.0 (0.1 – 555.7)	0.385
	malaria	13	7 (53.8)	1.4 (0.2 – 10.9)	0.732
Sources of care in the event of illness	#Hospital	177	47 (26.6)		0.177
	Self–medication and hospitals	52	15 (28.8)	6.2 (0.1 – 459.2)	0.404
	Self-medication	15	7 (46.7)	0.6 (0 – 20.5)	0.752
Self–medication: origin of care	#Pharmacy on the floor	1	0		0.975
	Approved pharmacy	4	1 (25.0)	0.3 (0 – 51.6)	0.648
	Traditional plants	139	68 (48.9)	0.5 (0 – 20.0)	0.726
Wastewater weir location	#Lost well	6	1 (16.7)		0.666
	In the street	22	6 (27.3)	4.9 (0.1 – 242.9)	0.425
	In the bush/landfill	216	62 (28.7)	1.5 (0.3 – 8.5)	0.634
Garbage spillway location	#In the bush/landfill	236	67 (28.4)		0.769
	Lost well	5	1 (20.0)	1.1 (0 – 35.3)	0.943
	Incineration	3	1 (33.3)	0.3 (0 – 41.0)	0.616
Mosquito net	#No	210	56 (26.7)	0.6 (0.1 – 3.0)	0.526
	Yes	34	13 (38.2)		
Net condition	#No mosquito net	34	13 (38.2)		0.183
	In good condition	148	33 (22.3)	0.4 (0.1 – 1.5)	0.198
	Faulty	62	23 (37.1)	0.2 (0 – 21.9)	0.790
Mosquito net used last night?	#No	190	53 (27.9)	1.2 (0.3 – 4.8)	0.790
	Yes	54	16 (29.6)		
Indoor mosquito control	#Timor and/or mosquito net	217	59 (27.2)		0.706
	Keeping the house clean	3	1 (33.3)	2.1 (0.3 – 14.4)	0.449
	Ventilator	9	4 (44.4)	2.2 (0 – 110.7)	0.694
	No provision	15	5 (33.3)	6.5 (0.3 – 143.1)	0.237
Last malaria care	#Self-medication	20	8 (40.0)		0.184
	Self–medication and hospital	31	13 (41.9)	0.4 (0.1 – 2.1)	0.248
	Hospital	193	48 (24.9)	1.7 (0.2 – 17.3)	0.674
Date of last hospital visit	#More than 2 years	8	1 (12.5)		0.681
	7 months to 2 years	39	15 (38.5)	1.6 (0 – 59.7)	0.800
	1–6 months	171	46 (26.9)	3.5 (0.1 – 101.1)	0.459
	Less than one month	26	7 (26.9)	3.3 (0.1 – 78.8)	0.468
Mosquito nuisance frequency	#Rare	44	7 (15.9)	0.2 (0 - 0.7)	0.017*
	Several times a day	200	62 (31.0)		

Mosquito development	#Ignorant people	54	29 (53.7)	2.5 (0.9 – 7.3)	0.096
	People who know	190	49 (25.8)		
Outdoor mosquito control	#No provision	69	23 (33.3)		0.755
	Keeping it clean	173	44 (25.4)	0	0.999
	Spraying	2	2 (100.0)	0	0.999
Presence of puddles	#No	52	12 (23.1)	0.5 (0.1 – 3.3)	0.496
	Yes	192	57 (29.7)		
Drinking water sources	#Other	28	9 (32.1)		0.651
	Domestic well	180	50 (27.8)	NA	0.999
	Domestic well and pumps	31	10 (32.3)	NA	0.999
	Pumps	5	0	NA	0.999
Water–related activities?	#No	69	15 (21.7)	0.4 (0.1 – 1.4)	0.129
	Yes	175	54 (30.9)		
Stream near the house?	#No	55	14 (25.4)	0.8 (0.1 – 4.7)	0.786
	Yes	189	55 (29.1)		
Do you sleep under a mosquito net?	#No mosquito net	34	13 (38.2)		0.759
	No	137	35 (47.9)	1.3 (0.3 – 5.2)	0.759
	Yes	73	21 (28.8)	0.9 (0 – 28.3)	0.946
Do you store water?	#No	241	68 (28.2)	1.3 (0.2 – 7.2)	0.779
	Yes	3	1 (33.3)		
Do you cover this water?	#No	217	62 (28.6)	1.1 (0.3 – 4.4)	0.941
	Yes	27	7 (25.9)		
Do you treat this water?	#No	31	8 (25.8)	NA	0.946
	yes	213	61 (28.6)		
Activities around the Bandama River	#No activity	207	62 (29.9)		0.304
	Fishing	14	0	0.1 (0 – 1.1)	0.059
	Swimming and other	17	5 (29.4)	0	0.998
	Vegetable and rice growing	6	2 (33.3)	0.1 (0 – 3.0)	0.197

N = number of people interviewed, **n** = number of people infected by malaria, **%** = proportion of people infected by malaria, **NA** = not applicable, **OR** = odds ratio, **CI 95%** = 95% confidence interval, (*) significant variables, and (**#**) reference category.
